# Temperature characteristics of fiber-optic shearer cables

**DOI:** 10.1371/journal.pone.0356684

**Published:** 2026-08-25

**Authors:** Feng Gao, Shutian Gong, Zifeng Liu, Lijuan Zhao, Jing Liu, Tianyi Zhang, Yang Liu, Feida Wang, Dongyang Wang, Tiangu Wu, Zhongjian Bai, Shuo Tian

**Affiliations:** 1 Changlong Cable Manufacturing Co., Ltd., Shandong Yankuang Group, Jining, Shandong, China; 2 School of Mechanical Engineering, Liaoning Technical University, Fuxin, Liaoning, China; 3 Liaoning Professional Technology Innovation Center of Hydraulic Transmission and Control, Fuxin, Liaoning, China; Zhejiang University, CHINA

## Abstract

During the reciprocating motion of a coal shearer, shearer cables are exposed to complex underground conditions, including high load current, repeated bending and dragging, and limited heat dissipation. These factors may lead to excessive conductor temperature and pose safety risks. However, the temperature response of embedded optical-fiber units in shearer cables under different motion states remains insufficiently clarified, especially the relationship between optical-fiber temperature and the temperatures of key cable components. To address this gap, an MCPT-1.9/3.3(3 × 120 + 1 × 70 + 4 × 10) shearer cable was taken as the engineering object, and three optical-fiber unit structures were designed and embedded to form three fiber-optic shearer cable configurations. Parametric models were developed using Rhino–Grasshopper, and COMSOL Multiphysics was used to analyze the effects of cable structure, load current, and ambient temperature under straight-line and bending motions. The results show that the maximum optical-fiber temperatures of the three structures are 57.8°C, 58.1°C, and 63.8°C under straight-line motion, increasing to 69.5°C, 70.1°C, and 77.4°C under bending motion. Optical-fiber temperature rises with increasing load current and ambient temperature. Least-squares fitting further establishes relationships between optical-fiber temperature and the temperatures of the power conductor, control conductor, polyimide buffer layer, and outer sheath. The main contribution of this study is to provide a quantitative basis for temperature monitoring and structural optimization of fiber-optic shearer cables under different operating conditions.

## 1. Introduction

Owing to the harsh underground working environment, shearer cables experience severe operating conditions during reciprocating motion on fully mechanized mining faces. These conditions include high-load operation, frequent bending and dragging, and limited heat dissipation. As a result, the cable is subjected to multiple thermal loads, including current-induced heating and ambient thermal effects. Long-term operation under such extreme conditions, combined with the lack of real-time feedback regarding the cable operating state, may lead to excessive temperature rise in the conductor core. In severe cases, this can accelerate insulation aging, induce short-circuit faults, interrupt mining operations, and reduce mining efficiency [1].

In recent years, extensive studies have been conducted worldwide on the temperature characteristics of fiber-optic cables. Abdelghani Matine et al. [2] investigated the influence of conductor failure on the thermal behavior of submarine fiber-optic power cables using the finite element method and employed optical fibers as sensors for structural health monitoring. Ahsan Ashfaq et al. [3] analyzed the temperature distribution of optical-fiber composite low-voltage cables when the current increased to the maximum capacity, selected an optimal heat-resistant layer for the optical-fiber unit, and used a BOTDA analyzer to monitor the internal cable temperature under normal and overload conditions, with the results validated against simulations. M. N. González-Cagigal et al. [4] proposed an equivalent thermal network model for three-core armored submarine cables. By adjusting the optical-fiber position in the equivalent circuit, the model accurately estimated conductor temperature using sensor measurements. Fu et al. [5] established a finite element model in COMSOL to investigate transient high-temperature issues in optical fibers during the operation of low-voltage composite fiber-optic cables and optimized the cable structure and manufacturing process based on simulation and experimental results. Chen Yu et al. [6] proposed an optimized model for optical-fiber composite low-voltage cables. By varying the optical-unit position, heat-resistant material, and layer thickness, COMSOL simulations were used to compare the temperature distributions before and after structural optimization. The results showed that the optimized structure effectively reduced the influence of electrothermal effects on the optical fibers. Liu et al. [7] proposed an XLPE cable insulation fault detection method based on optical-fiber temperature sensors. Models representing different insulation degradation levels were established, and the relationship between cable surface temperature rise and insulation failure degree was obtained. Experiments based on distributed optical-fiber temperature sensing verified the effectiveness and accuracy of the method. Lian Guo [8] addressed the poor heat resistance of optical units by optimizing the design of optical-fiber composite low-voltage cables and independently developing a heat-resistant optical unit for OPLC, which effectively reduced the impact of transient high temperature on optical-fiber transmission. Ti Heng [9] studied the temperature distribution of cables under normal operation, insulation aging, and fault conditions through simulation. The temperature-field characteristics under different operating states were obtained, providing theoretical and data support for cable condition monitoring using distributed optical-fiber temperature sensing. Li Yunbo et al. [10] established an electrical–thermal–fluid coupled finite element model for three-core composite fiber-optic submarine cables, derived the relationship between conductor and optical-fiber temperatures, and analyzed the influence of different cable dimensions. Their results indicated that, at the same conductor temperature, a larger conductor cross-sectional area leads to a higher optical-fiber temperature. Zhao L. et al. [11] investigated the effects of ambient temperature and load current on cable temperature and accurately predicted shearer cable temperature using a bidirectional long short-term memory neural network, with a maximum prediction error below 2%.

Although previous studies have provided useful methods for cable temperature-field simulation, optical-fiber temperature monitoring, structural optimization, and temperature prediction, their applicability to coal shearer cables remains limited. Most existing studies on fiber-optic cables have focused on submarine cables, low-voltage optical-fiber composite cables, XLPE power cables, or OPLC systems. These cables differ significantly from coal shearer trailing cables in structural configuration, operating environment, and motion state. In particular, the reciprocating bending and dragging motion of coal shearer cables in underground mines has rarely been considered in existing studies on the temperature characteristics of fiber-optic cables. Meanwhile, previous studies on coal shearer cables have mainly focused on temperature prediction or multi-physical-field behavior of conventional cable structures, whereas the integration of embedded optical-fiber units into shearer cables and the resulting temperature response of fiber-optic shearer cable structures remain insufficiently investigated.

Therefore, the research gap addressed in this study is the lack of a quantitative understanding of the thermal response of embedded optical-fiber units in coal shearer cables under different motion states and operating conditions. Specifically, it remains unclear how the arrangement and placement of optical-fiber units affect the optical-fiber temperature, and how the measured optical-fiber temperature can be related to the temperatures of key internal cable components, such as the power unit conductor, control unit conductor, polyimide buffer layer, and outer sheath.

To address this gap, this study takes an MCPT-1.9/3.3(3 × 120 + 1 × 70 + 4 × 10) coal shearer cable as the engineering object and designs three embedded optical-fiber unit configurations. Parametric three-dimensional models of the fiber-optic shearer cables are constructed using Rhino–Grasshopper, and COMSOL Multiphysics is used to simulate the temperature fields under straight-line and bending motions. The effects of optical-fiber unit structure, load current, and ambient temperature on the internal optical-fiber temperature are analyzed. Furthermore, the least-squares method is used to establish functional relationships between the optical-fiber temperature and the temperatures of key cable components. The novelty of this work lies in the structural design of embedded optical-fiber units for coal shearer cables and the quantitative evaluation of their temperature response under both straight-line and bending motions. The objective is to provide a quantitative basis for temperature monitoring and structural optimization of fiber-optic coal shearer cables under complex underground operating conditions.

## 2. Model construction of the fiber-optic shearer cable

### 2.1. Design of the optical-fiber unit

The optical-fiber unit designed in this study consists of four optical fibers, together with their buffer layer and sheath. Two fiber arrangements are considered: a 1 + 3 parallel arrangement and a 1 + 3 stranded arrangement. The 1 + 3 parallel arrangement consists of one central optical fiber and three parallel optical fibers uniformly distributed around it. The 1 + 3 stranded arrangement consists of one central optical fiber and three outer optical fibers stranded around it. Based on the Rhino-Grasshopper parametric modeling method, different reference lines are used to construct the above arrangements, resulting in three optical-fiber unit structural models.

**Fig 1:**
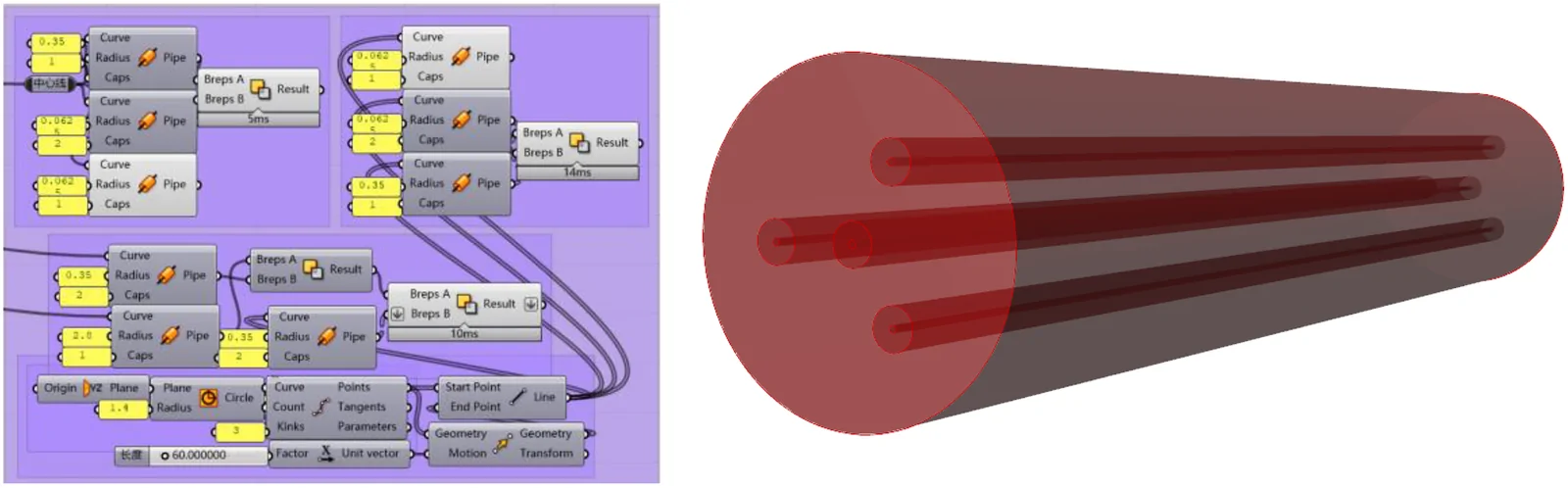
Modeling workflow and three-dimensional model of the first optical-fiber unit structure.

In the first optical-fiber unit structure, the internal optical fibers adopt a 1 + 3 parallel arrangement, and the model is constructed using the central line as the reference line. The modeling workflow and three-dimensional model of the first optical-fiber unit structure are shown in Fig 1.

**Fig 2:**
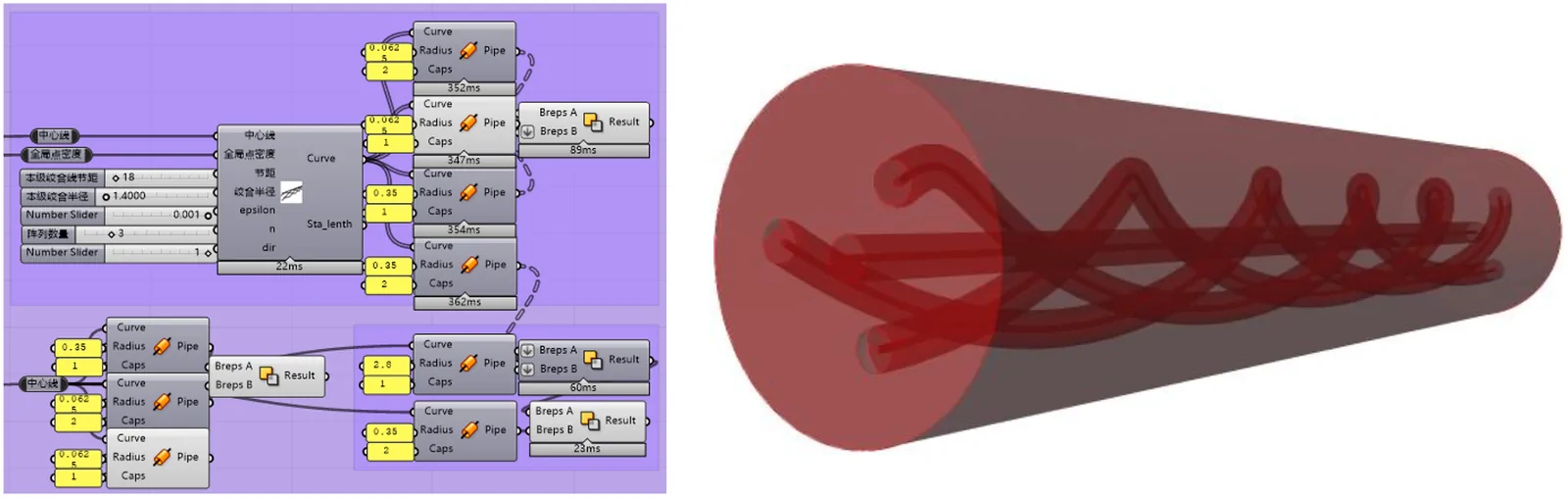
Modeling workflow and three-dimensional model of the second optical-fiber unit structure.

In the second optical-fiber unit structure, the internal optical fibers adopt a 1 + 3 stranded arrangement, with the central line used as the reference line. The modeling workflow and three-dimensional model of the second optical-fiber unit structure are shown in Fig 2.

**Fig 3:**
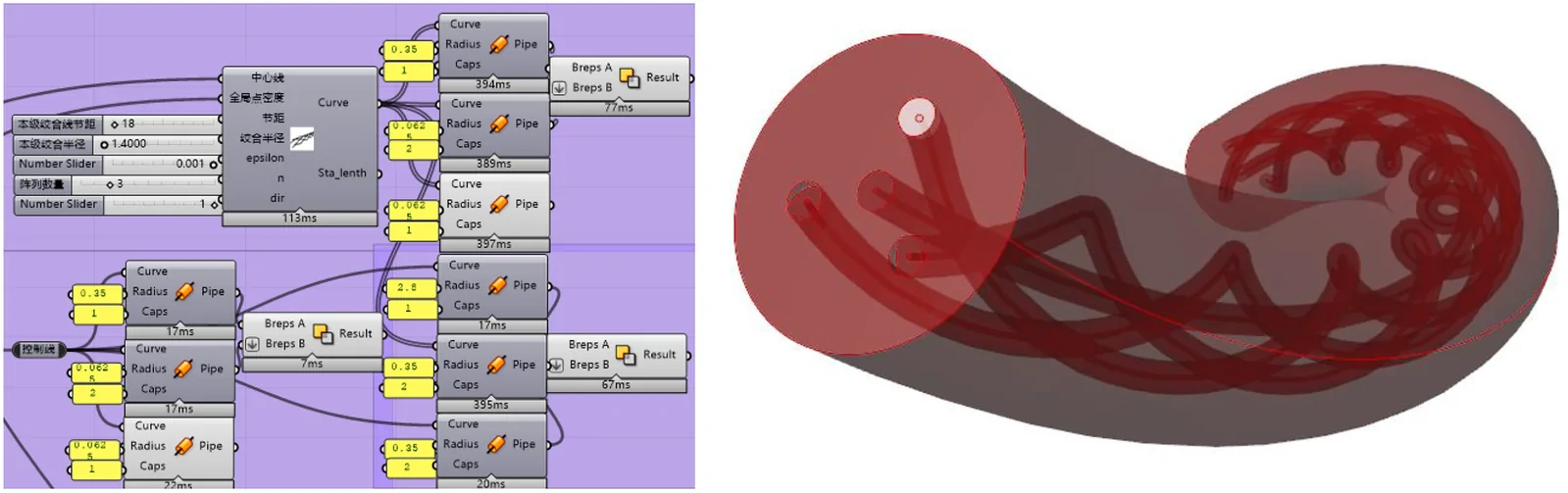
Modeling workflow and three-dimensional model of the third optical-fiber unit structure.

In the third optical-fiber unit structure, the internal optical fibers also adopt a 1 + 3 stranded arrangement, but the model is constructed using a secondary helical line as the reference line. The modeling workflow and three-dimensional model of the third optical-fiber unit structure are shown in Fig 3.

**Fig 4:**
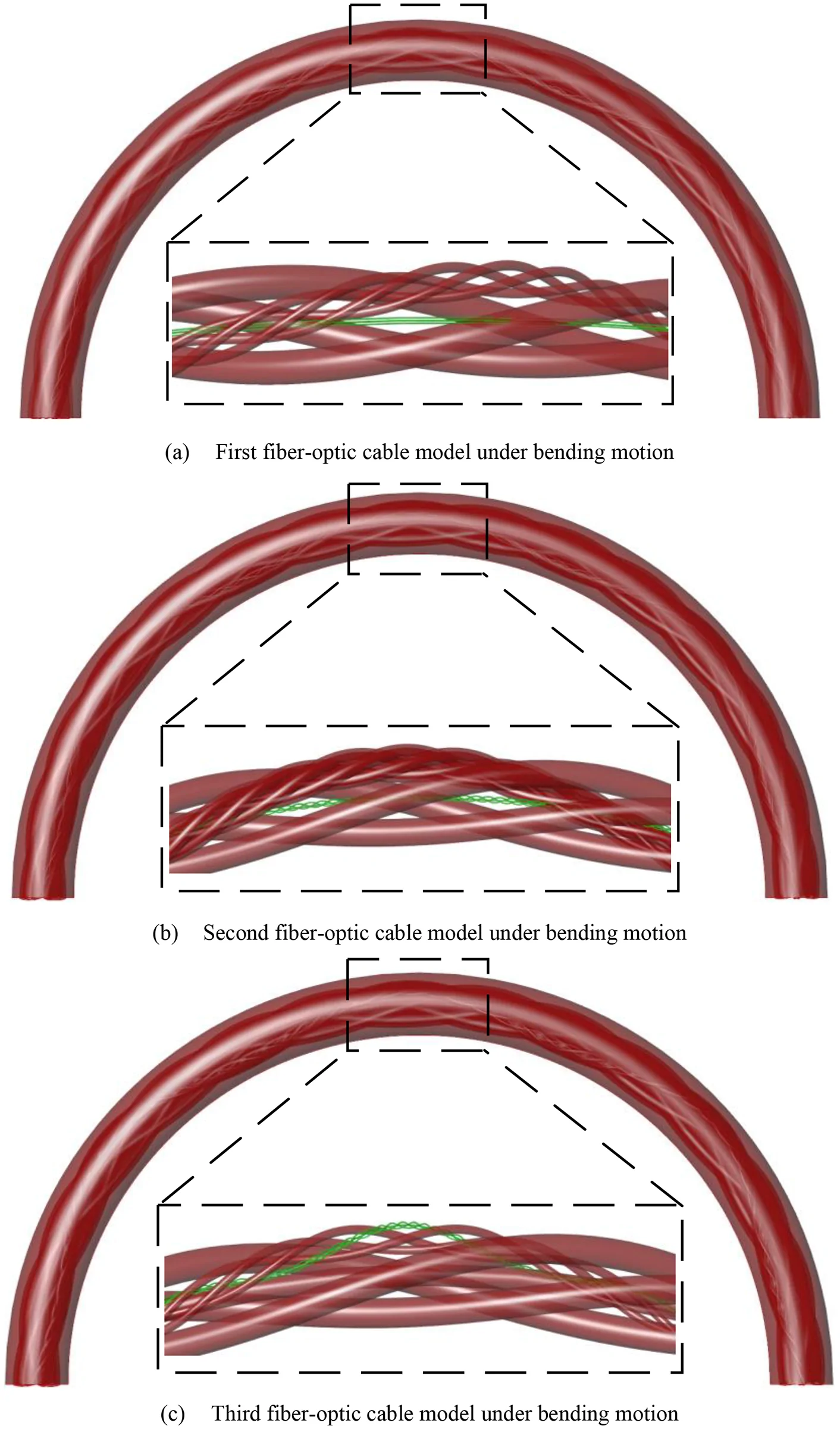
Three-dimensional models of the three fiber-optic cable structures under bending motion. (a) First fiber-optic cable model under bending motion, (b) Second fiber-optic cable model under bending motion. (c) Third fiber-optic cable model under bending motion.

When constructing the fiber-optic cable model under bending motion, the modeling procedure is generally consistent with that used for straight-line motion. The bending radius, midpoint, and two endpoints of the cable are first defined. A curved centerline corresponding to the bending motion is then generated and used as the reference centerline. By connecting the relevant functional modules, the three-dimensional models of the fiber-optic cable under bending motion are established, as shown in Fig 4.

### 2.2. Design of the fiber-optic shearer cable

**Fig 5:**
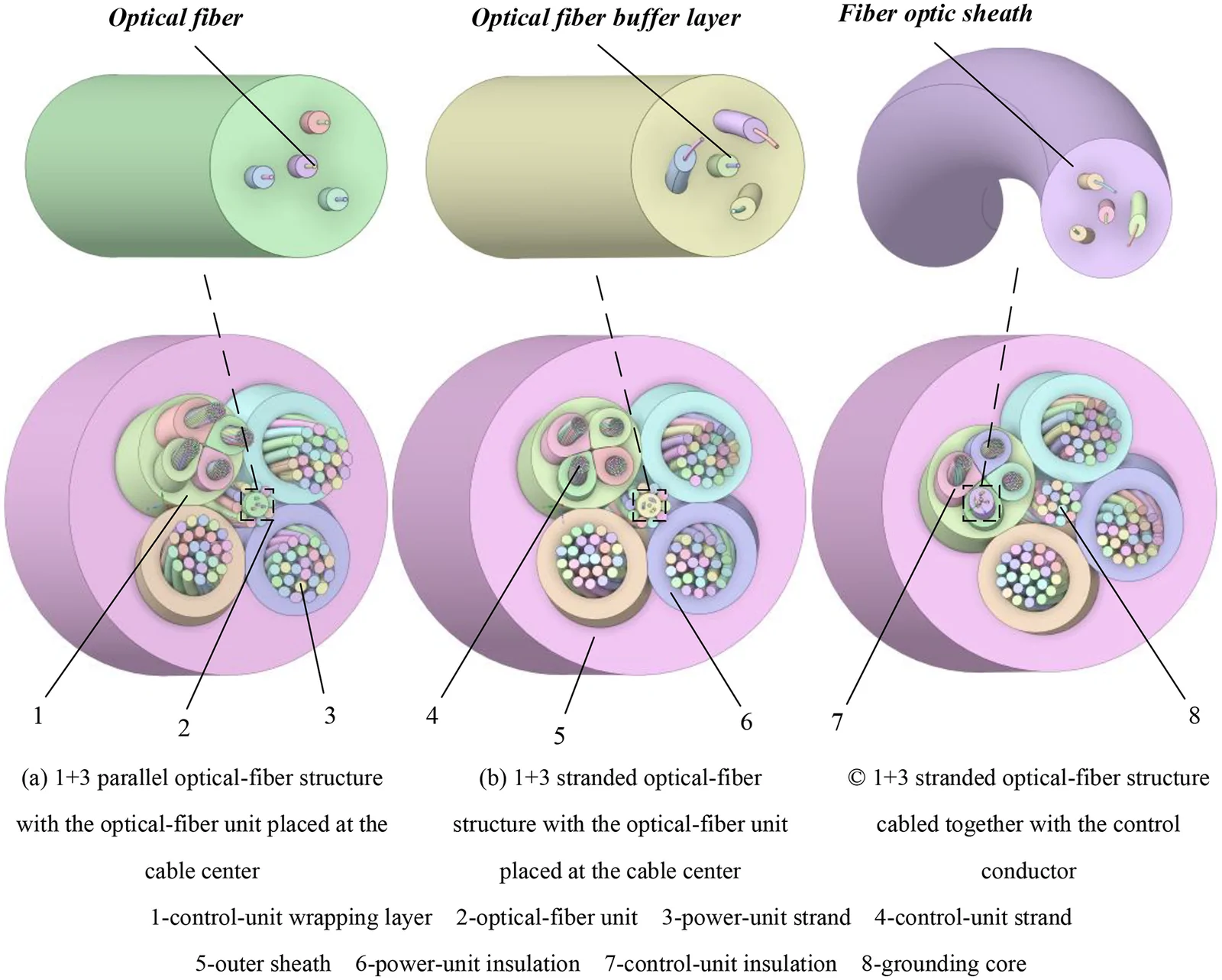
Three-dimensional models of the three fiber-optic shearer cable structures. (a) 1 + 3 parallel optical-fiber structure with the optical-fiber unit placed at the cable center, (b) 1 + 3 stranded optical-fiber structure with the optical-fiber unit placed at the cable center, © 1 + 3 stranded optical-fiber structure cabled together with the control conductor. 1-control-unit wrapping layer, 2-optical-fiber unit, 3-power-unit strand, 4-control-unit strand, 5-outer sheath, 6-power-unit insulation, 7-control-unit insulation, 8-grounding core.

The internal geometric structure of the shearer cable is complex and exhibits a layered helical stranding arrangement. The project team established its parametric three-dimensional model using Rhino–Grasshopper [12,13]. The designed optical-fiber units were embedded inside the shearer cable to form three fiber-optic shearer cable structures. In the first two structures, the optical-fiber unit was placed at the cable center, with the grounding cores arranged around it. In the third structure, the optical-fiber unit was placed at the position of the control unit conductor and cabled together with it. The three-dimensional models of the three fiber-optic shearer cable structures are shown in Fig 5. The modeling parameters and material types of the fiber-optic shearer cable are listed in Table 1.

**Table 1:** Modeling parameters and material types of the fiber-optic shearer cable.

No.	Structure	Material	Outer diameter/mm
1	Optical fiber	Silica	0.125
2	Optical-fiber buffer layer	Polyimide	0.7
3	Optical-fiber sheath	EPDM rubber	5.6
4	Grounding core	Tinned copper	10.9
5	Control unit conductor	Tinned copper	4.3
6	Power unit conductor	Tinned copper	15.28
7	Power-core strand	Tinned copper	2.48
8	Power-unit insulation	EPDM rubber	24.5
9	Control-unit insulation	EPDM rubber	8.72
10	Outer sheath	Chloroprene rubber	76

### 2.3. Electrothermal simulation model and parameter settings

To ensure the accuracy of the temperature-field simulation, an electrothermal coupling method was adopted for the numerical analysis of the fiber-optic shearer cable. After importing the constructed cable model into COMSOL, operations such as creating work planes, partitioning domains, and removing selected solids were performed on the cable end face to improve mesh quality and simulation accuracy. According to the material characteristics of the different cable structures, copper was assigned to the power-core, control-core, and grounding-core conductors. The corresponding materials were assigned to the optical fiber, optical-fiber buffer layer, insulation layers, and outer sheath, as listed in Table 2.

**Table 2:** Material parameters for temperature-field simulation of the fiber-optic cable.

Material	Density/kg·m^−3^	Thermal expansion coefficient/ K^−1^	Relative permittivity	Thermal conductivity/ W·m^−1^·K^−1^	Heat capacity at constant pressure/ J·kg·K^−1^
Control unit conductor	8960	1.7 × 10^−5^	1	400	385
Power unit conductor	7766.8	1.7 × 10^−5^	1	400	385
Grounding-core conductor	7198.5	1.7 × 10^−5^	1	400	385
Silica	2203	5.5 × 10^−7^	3.75	1.38	703
EPDM rubber	1250	2.3 × 10^−4^	2.5	0.24	2090
Polyimide	1250	2.3 × 10^−4^	2.5	0.24	1400
Chloroprene rubber	1550	2.3 × 10^−4^	6.7	0.19	2090

According to the coal industry standard MT818.1-2009, C*ables for Coal Mines-Part 1: General Requirements for Mobile Flexible Cables* [14], current excitations were applied to the three power cores, four control cores, and grounding-core conductors of the fiber-optic cable [15]. The current of the power cores was set to 295 A, that of the control cores to 63 A, and that of the grounding core to 0 A. When alternating current is applied to the fiber-optic shearer cable, heat losses are generated in the internal conductors and insulating dielectric materials. Conductor losses are mainly caused by AC resistance, the skin effect, and the proximity effect, whereas dielectric losses are related to voltage, capacitance, and the dielectric loss angle. By assigning reasonable current loads and material parameters in COMSOL, the temperature-field distribution of the fiber-optic shearer cable can be obtained through electrothermal coupling calculations, allowing the actual operating state of the cable to be simulated more accurately.

## 3. Numerical analysis of the internal optical-fiber temperature field

**Fig 6:**
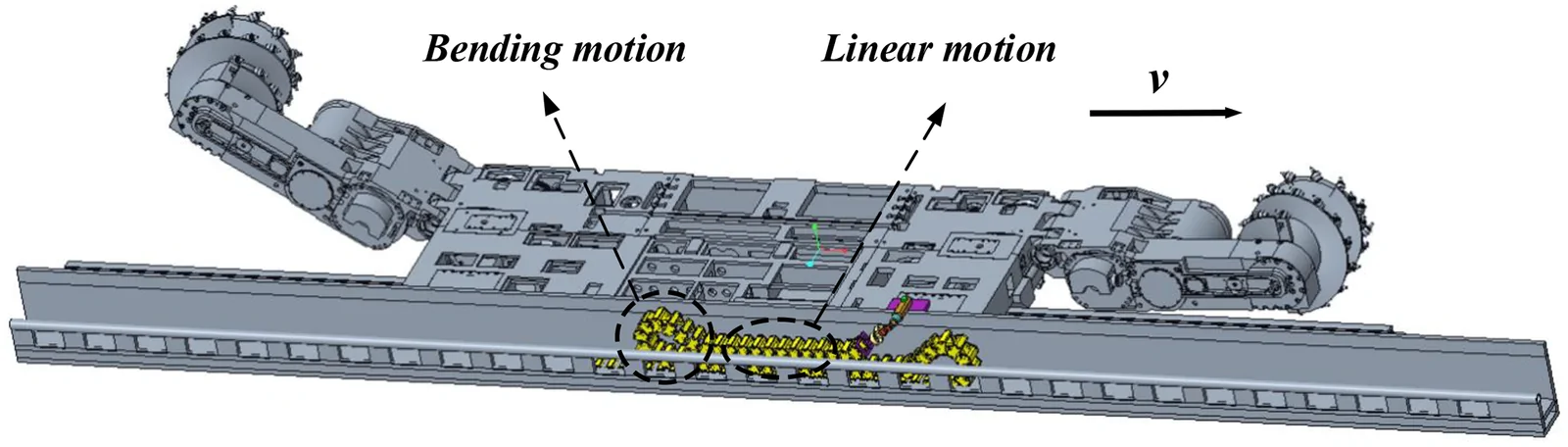
Schematic diagram of shearer cable operating states.

During operation, the fiber-optic shearer cable undergoes both straight-line and bending motions. The operating states of the shearer cable are illustrated in Fig 6. Numerical simulations were carried out for the three fiber-optic shearer cable models under different motion states, load currents, and ambient temperatures to investigate their temperature variation characteristics.

### 3.1. Numerical simulation setup

#### 3.1.1. Grid independence analysis.

Owing to the complex helical structure of the fiber-optic cable, mesh generation for the internal components is computationally demanding. A finer mesh generally improves numerical accuracy but significantly increases the computational cost, whereas a coarser mesh reduces the solution time but may introduce larger numerical errors. Therefore, a mesh-independence analysis was conducted to achieve an appropriate balance between computational accuracy and efficiency. Because the same cable geometry was used for the straight-line and bending configurations, the second fiber-optic cable structure under the straight-line configuration was selected for the mesh-independence analysis to improve computational efficiency.

**Fig 7:**
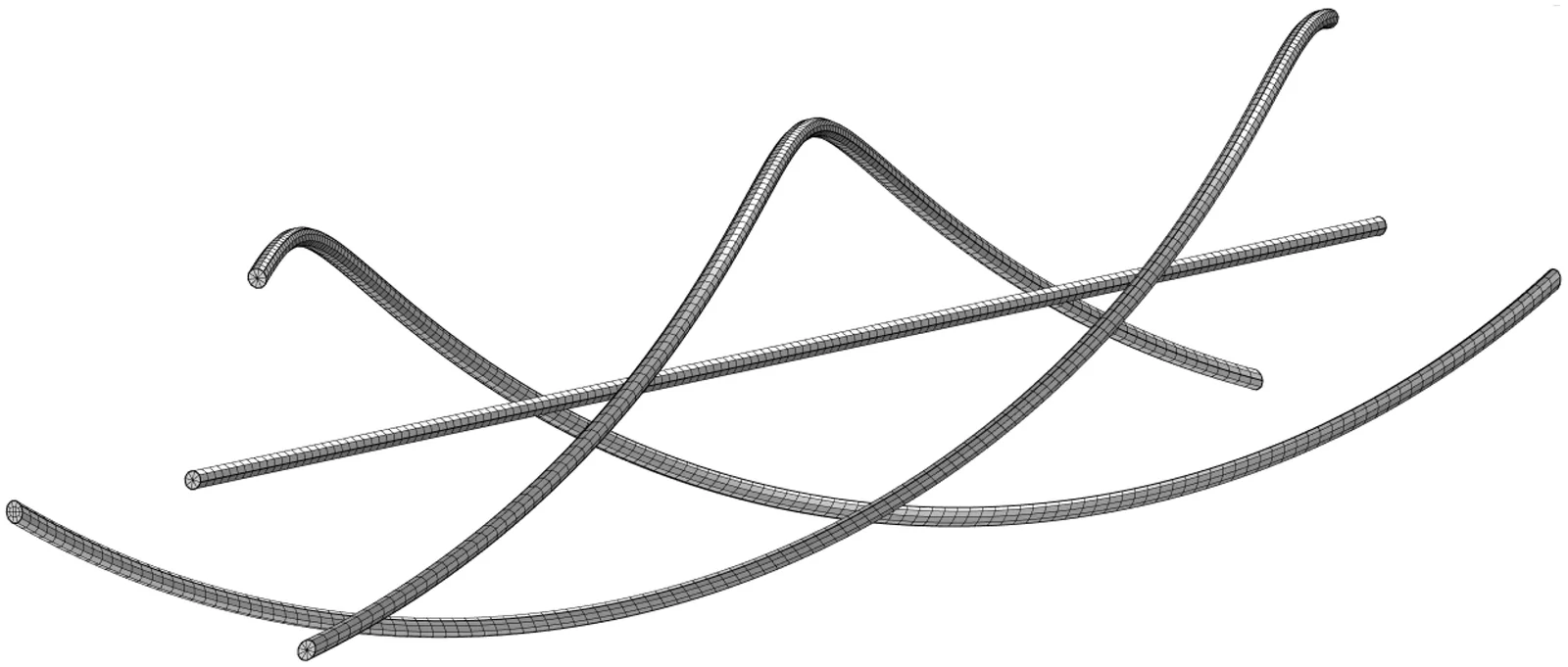
Meshing of optical fiber structure.

As shown in Table 3, the maximum optical-fiber temperature gradually increases and approaches a stable value as the mesh is refined. Using Model 5 as the reference, the relative errors of Models 1–4 are −5.99%, −3.25%, −1.71%, and −0.51%, respectively. Model 4 was therefore selected for the subsequent simulations because it provides sufficient accuracy while avoiding the excessive computational cost associated with further mesh refinement. The mesh division of fiber structures is shown in Fig 7.

**Table 3:** Mesh independence analysis of the cable.

Mesh model	Model 1	Model 2	Model 3	Model 4	Model 5
Number of edge units	15158	18536	22471	26202	30156
Number of boundary units	325846	390117	465418	546763	625913
Number of domain units	2458493	3158897	3921658	4645706	5381658
Maximum optical-fiber temperature (°C)	54.9	56.5	57.4	58.1	58.4
Error (%)	−5.99	−3.25	−1.71	−0.51	—

#### 3.1.2. Boundary conditions.

According to the coal industry standard MT 818.4–2009, *Cables for Coal Mines-Part 4* [16], the minimum bending radius of a shearer cable during operation shall be six times its outer diameter. In this study, a 1500 mm fiber-optic cable structure was established based on an MCPT-1.9/3.3(3 × 120 + 1 × 70 + 4 × 10) shearer cable. The outer diameter of the cable was 76 mm, corresponding to a bending radius of 456 mm.

Copper was selected as the conductor material for all cores of the fiber-optic shearer cable, while silica was used as the optical-fiber material. The optical-fiber buffer layer was made of polyimide, the insulation layers were composed of ethylene–propylene–diene monomer (EPDM) rubber, and the outer sheath was made of chloroprene rubber. The material properties of each cable component are listed in Table 4.

**Table 4:** Material parameters of temperature field of optical fiber cable.

Material	Density kg/m^3^	Coefficient of thermal expansion 1/K	Relative permittivity	Thermal conductivity W/(m·K)	Heat capacity at constant pressureJ/(kg·K)
Control unit conductor	8960	1.7 × 10^−5^	1	400	385
Power unit conductor	7766.8	1.7 × 10^−5^	1	400	385
Ground core conductor	7198.5	1.7 × 10^−5^	1	400	385
Silicon dioxide	2203	5.5 × 10^−7^	3.75	1.38	703
EPDM rubber	1250	2.3 × 10^−4^	2.5	0.24	2090
polyimide	1250	2.3 × 10^−4^	2.5	0.24	1400
Chloroprene rubber	1550	2.3 × 10^−4^	6.7	0.19	2090

**Fig 8:**
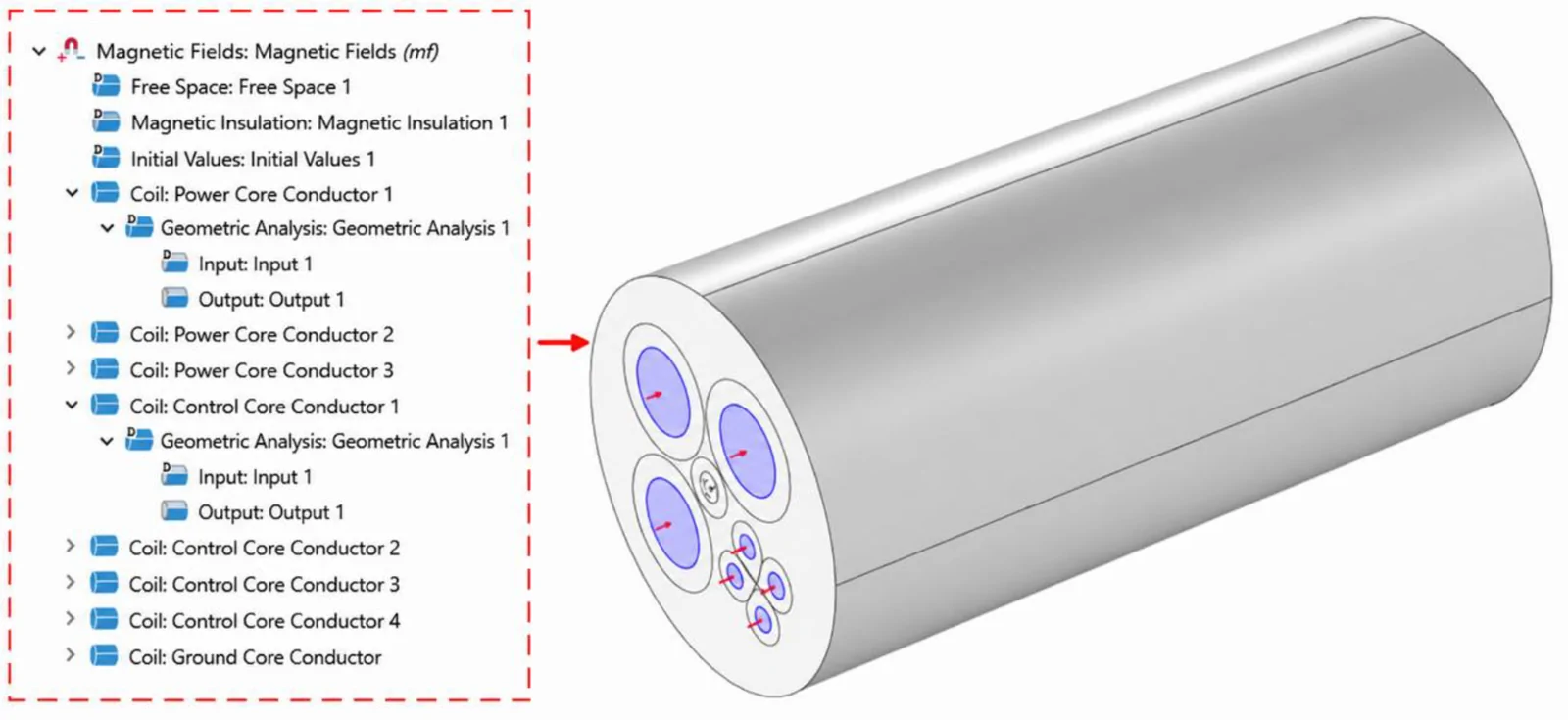
Current excitation applied to the conductor.

According to the requirements of the coal industry standard MT 818.1–2009, *Cables for Coal Mines-Part 1*, the load current of each conductor in a mobile flexible cable is related to its nominal cross-sectional area. The allowable load currents for conductors with different cross-sectional areas are listed in Table 5. In temperature-field simulations of the fiber-optic cable, the root mean square (RMS) current is generally adopted as the applied excitation because it accurately represents the heat generated in the conductor and surrounding media during current transmission. Eight coil domains were defined in the model tree, corresponding to the three power unit conductors, four control unit conductors, and one ground-core conductor of the fiber-optic cable. In the geometric analysis settings of the coil feature, current excitations together with the corresponding input and output directions were assigned to the power-core, control-core, and ground-core conductors, respectively [11,15]. The current amplitudes were set to 295 A, 63 A, and 0 A for the power-core, control-core, and ground-core conductors, respectively. The conductor current excitations are illustrated in Fig 8.

**Table 5:** Current load of conductors with different cross-sectional areas.

Conductor type	Cross-sectional area (mm^2^)	Phase angle (°)	Frequency (Hz)	Load current (A)
Power unit conductor 1	120	0	50	295
Power unit conductor 2	120	120	50	295
Power unit conductor 3	120	−120	50	295
Ground conductor core	70	−	−	0
Control conductor core	10	−	50	63

The solution settings were configured before numerical simulation. The operating frequency was set to 50 Hz, and the MUMPS solver was employed for computation. The maximum number of iterations was specified as 25. The remaining parameters were determined according to the specific simulation requirements and were adjusted as needed during the simulation process.

#### 3.1.3. Heat loss.

When alternating current is applied to the shearer fiber-optic cable, additional losses are generated within the cable components, including the copper conductors and insulating media. These losses contribute significantly to heat generation inside the cable. In particular, conductor losses and dielectric losses increase the thermal load and consequently affect the temperature distribution of the cable. The associated heat losses can be calculated using the method specified in the IEC 60287 standard [17].

(1) Internal conductor loss of optical cables

Due to the resistance of the conductor, heat loss occurs when current passes through the conductor. The expression for heat loss per unit length of the conductor is


$$\begin{array}{c} W_{\text{1}}=I^{\text{2}}R_{ac} \end{array}$$ (1)

In the formula, *I* is the current flowing through the conductor, A; *R*_*ac*_ is the AC resistance per unit length of conductor, Ω/m.

When a conductor operates for a long time at the highest allowable temperature, the AC resistance expression per unit length of conductor is


$$\begin{array}{c} R_{ac}=R_{t}\left(1+y_{s}+y_{p}\right) \end{array}$$ (2)

In the formula, *R*_*t*_ is the DC resistance per unit length of the conductor, Ω/m; *y*_*s*_ is the skin effect coefficient; *y*_*p*_ is the proximity effect coefficient.

When the conductor temperature is *θ*_*c*_, the expression for DC resistance is:


$$\begin{array}{c} R_{t}=R_{20}\left[1+\alpha_{20}\left(\theta_{c}-20\right)\right] \end{array}$$ (3)

In the formula, *R*_20_ is the DC resistance of the conductor per unit length at 20°C, Ω/m; *α*_20_ is the temperature coefficient of the conductor core material at 20°C, 1/°C; *θ*_*c*_ is the temperature of the optical cable core, °C

The skin effect refers to the phenomenon that, when alternating current flows through a conductor, the current tends to concentrate near the conductor surface due to the electromagnetic field. As a result, the effective cross-sectional area available for current conduction decreases, leading to an increase in the effective resistance and a reduction in current-carrying capability [18]. The skin-effect coefficient of the fiber-optic cable can be expressed as follows:


$$\begin{array}{c} y_{s}=\frac{x_{s}^{\text{4}}}{192+0.8x_{s}^{\text{4}}} \end{array}$$ (4)

$$\begin{array}{c} x_{s}=\frac{8\pi f}{R_{T}}\times 10^{-7}k_{s} \end{array}$$ (5)

In the formula, *f* is the current frequency, Hz; *k*_*s*_=1.0.

The proximity effect refers to the phenomenon where multiple conductors are affected by current flow, causing current to concentrate on the conductor surface and resulting in uneven current distribution and thus increased resistance [19]. The expression for the proximity effect coefficient of optical cables is


$$\begin{array}{c} y_{p}=\frac{x_{p}^{\text{4}}}{192+0.8x_{p}^{\text{4}}}{\left(\frac{d_{c}}{s}\right)}^{\text{2}}\times \left[0.312\left(\frac{d_{c}}{s}\right)+\frac{1.18}{\frac{x_{p}^{\text{4}}}{192+0.8x_{p}^{\text{4}}}+0.27}\right] \end{array}$$ (6)

$$\begin{array}{c} x_{p}=\frac{8\pi f}{R_{t}}\times 10^{-7}k_{p} \end{array}$$ (7)

In the formula, *d*_*c*_ is the conductor core diameter, mm; *s* is the distance between each axis of the conductor, mm; *k*_*p*_ = 1.0.

(2) Loss of the insulation medium for optical cables

$$\begin{array}{c} W_{d}=\omega \cdot C\cdot U^{\text{2}}\cdot tan\delta \end{array}$$ (8)

$$\begin{array}{c} C=\frac{\varepsilon }{18ln\left(\frac{D_{i}}{d_{c}}\right)}\times 10^{-9} \end{array}$$ (9)

In the formula, *ω* is the AC voltage angular frequency; *C* is the capacitance per unit length of optical cable, F/m; *U* is the phase voltage, V; tanδ is the loss coefficient of the dielectric insulation;δ is the dielectric loss angle, °; *ε* is the dielectric constant of the insulating material; *D*_*i*_ is the outer diameter of insulation, mm.

### 3.2. Effect of different fiber-optic cable structures on optical-fiber temperature

#### 3.2.1. Optical-fiber temperature characteristics under straight-line motion.

**Fig 9:**
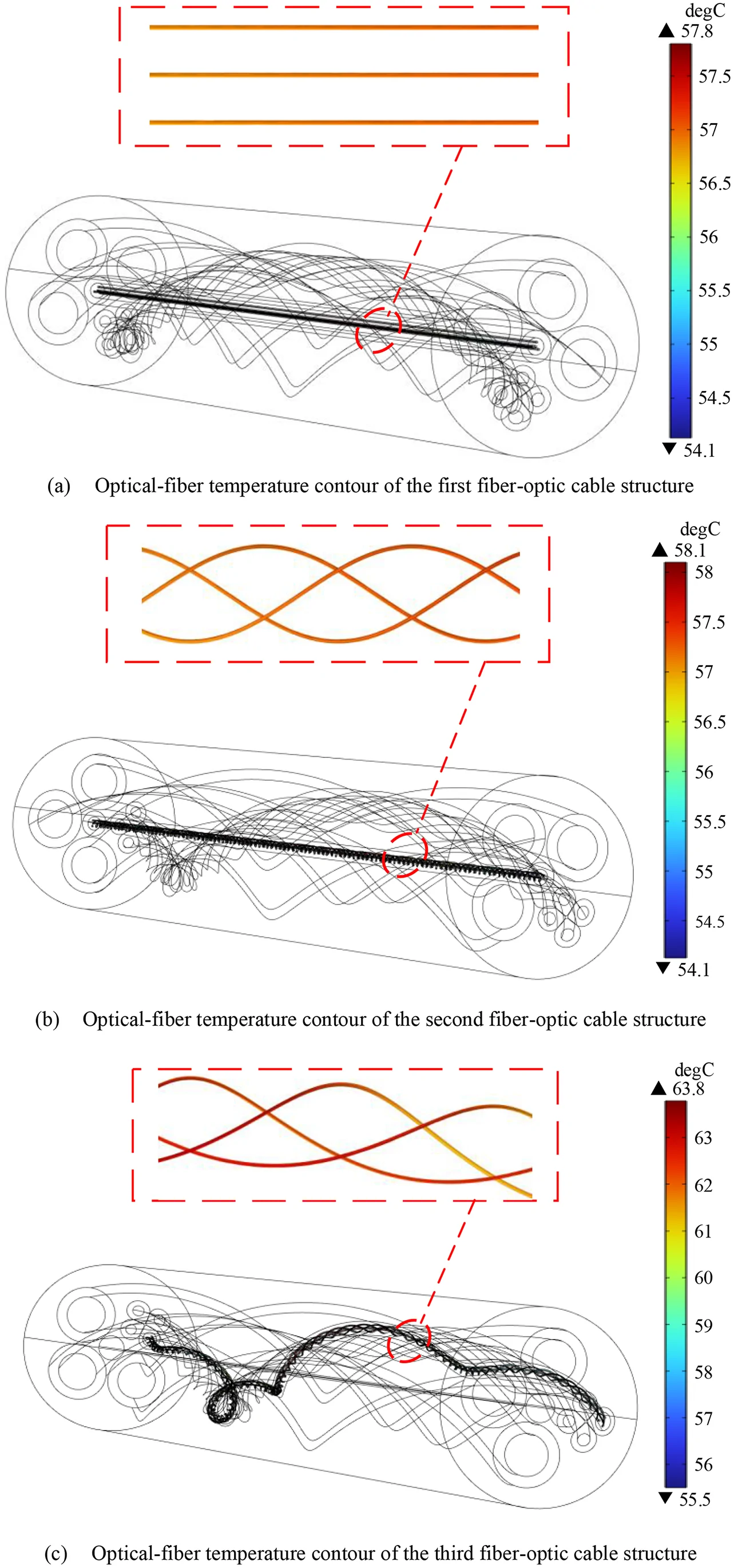
Optical-fiber temperature contours of the three fiber-optic cable structures under straight-line motion. (a) Optical-fiber temperature contour of the first fiber-optic cable structure. (b) Optical-fiber temperature contour of the second fiber-optic cable structure. (c) Optical-fiber temperature contour of the third fiber-optic cable structure.

Under an ambient temperature of 20 °C, with the load currents of the power-unit and control unit conductors set to 295 A and 63 A, respectively, the three fiber-optic cable structures under straight-line motion were simulated. The optical-fiber temperature contours are shown in Fig 9. As shown in Fig 9(a) and 9(b), when the optical-fiber units are both placed at the cable center, changing only the fiber arrangement from a 1 + 3 parallel distribution to a stranded distribution results in maximum optical-fiber temperatures of 57.8 °C and 58.1 °C, respectively. The difference is small, and the temperature-field distributions are generally consistent. This is because the spatial distances between the optical fibers and the conductor heat sources are similar in the two structures. The local geometric arrangement has a limited influence on the heat-conduction path; therefore, the stranding mode of the optical fibers has no significant effect on temperature rise. A comparison of Fig 9(b) and 9(c) indicates that, when the optical fibers adopt the same stranded arrangement, the placement position of the optical-fiber unit has a pronounced effect on temperature distribution. After the optical-fiber unit is moved from the cable center to the position where it is cabled together with the control unit conductor, the maximum temperature increases from 58.1 °C to 63.8 °C. This is because the latter arrangement places the optical fibers closer to the conductor heat source, shortens the heat-conduction path, and intensifies heat accumulation in the optical-fiber region, thereby significantly increasing the optical-fiber temperature.

#### 3.2.2. Optical-fiber temperature characteristics under bending motion.

**Fig 10:**
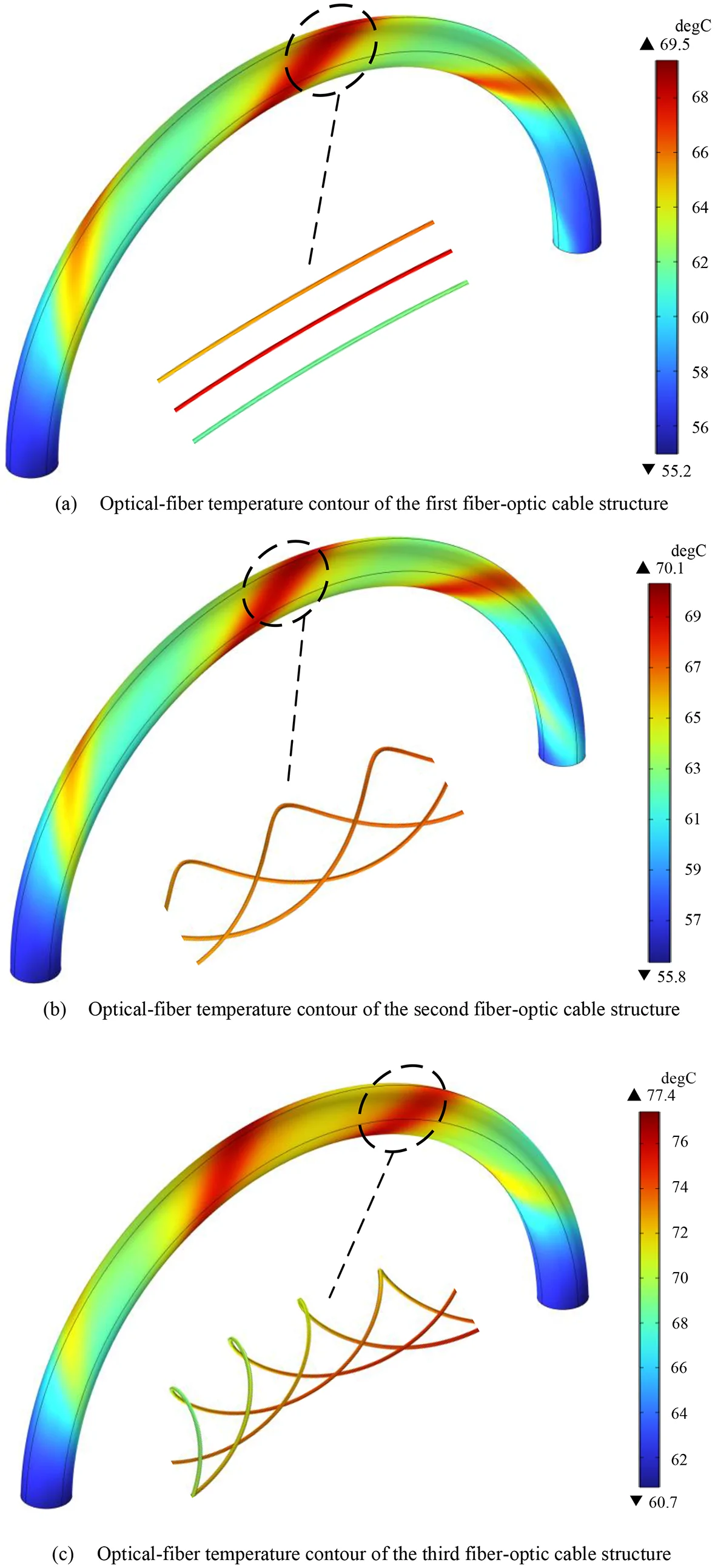
Optical-fiber temperature contours of the three fiber-optic cable structures under bending motion. (a) Optical-fiber temperature contour of the first fiber-optic cable structure. (b) Optical-fiber temperature contour of the second fiber-optic cable structure. (c) Optical-fiber temperature contour of the third fiber-optic cable structure.

During operation, the fiber-optic shearer cable undergoes repeated straight-line and bending motions. In the present numerical analysis, the bending state was represented by reconstructing the cable geometry with the prescribed bending radius, while the material properties, electrical loads, and thermal boundary conditions were kept unchanged. Mechanical deformation, inter-component contact, frictional heat generation, and deformation-induced electrical-resistance changes were not included in the model. Therefore, the comparison between the straight-line and bending configurations reflects the influence of geometric configuration on the electrothermal field. Numerical simulations were conducted for the three cable structures under the same boundary conditions, and the corresponding optical-fiber temperature contours are shown in Fig 10. The extracted temperatures under straight-line and bending configurations are summarized in Table 6.

**Table 6:** Optical-fiber temperatures of the three fiber-optic cable structures under straight-line and bending motions.

Motion state	Fiber-optic shearer cable structure	Internal optical-fiber temperature/°C
Straight-line motion	First structure	57.8
	Second structure	58.1
	Third structure	63.8
Bending motion	First structure	69.5
	Second structure	70.1
	Third structure	77.4

As shown in Fig 10 and Table 6, the optical-fiber temperatures of the three cable structures under the curved configuration were 69.5 °C, 70.1 °C, and 77.4 °C, respectively. The temperature trend was generally consistent with that observed under the straight-line configuration. Under the rated-load condition, the curved configurations produced higher maximum optical-fiber temperatures than the corresponding straight-line configurations. Because mechanical deformation, inter-component contact, frictional heating, and deformation-induced resistance changes were not included in the model, these differences reflect only the influence of geometric configuration and the resulting heat-transfer paths in the electrothermal field.

Because the optical-fiber units in the first and second fiber-optic cable structures are both located at the cable center, the optical-fiber temperatures under straight-line and bending motions differ only slightly. This indicates that the fiber arrangement has no significant effect on the temperature field. In contrast, in the third structure, the optical-fiber unit is positioned close to the control unit conductor, leading to a marked increase in optical-fiber temperature. Therefore, the placement position of the optical-fiber unit is the dominant factor affecting temperature-field distribution. To further analyze the effects of load current and ambient temperature on optical-fiber temperature while reducing repeated calculations, the second fiber-optic cable structure, which exhibits a representative temperature response, was selected for subsequent analysis.

### 3.3. Effect of load current on optical-fiber temperature

**Fig 11:**
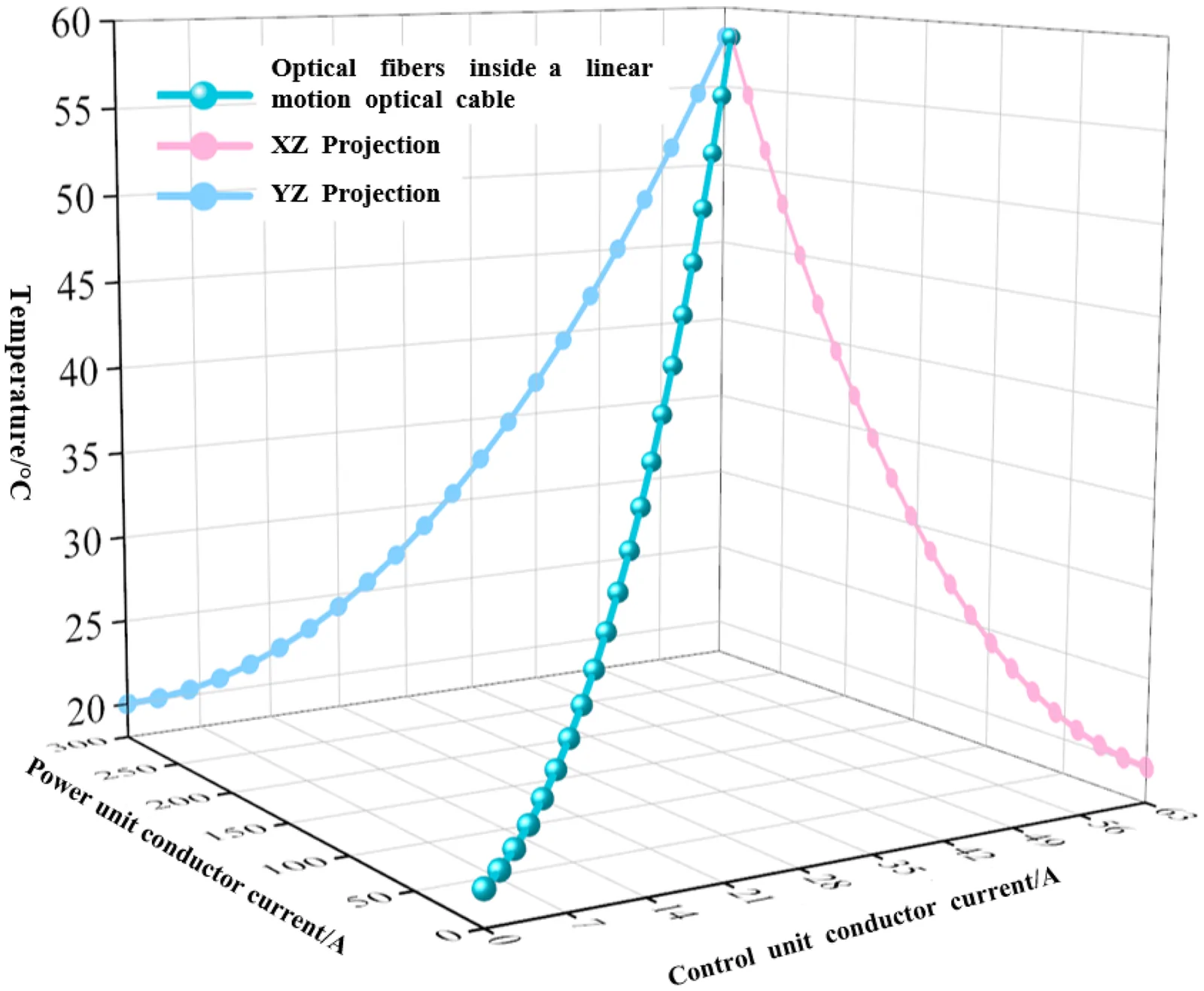
Variation curve of optical-fiber temperature with load current under straight-line motion.

**Fig 12:**
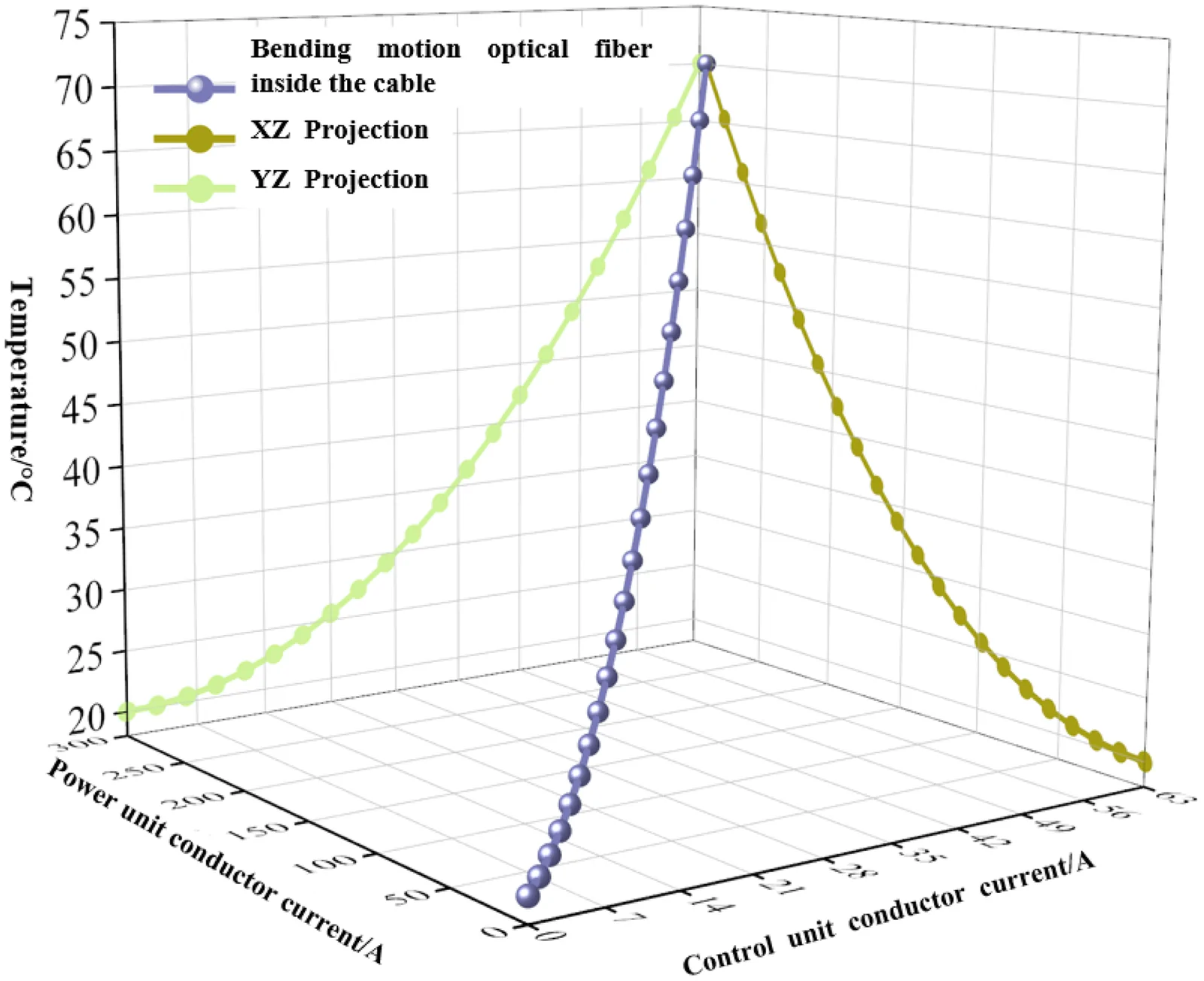
Variation curve of optical-fiber temperature with load current under bending motion.

To reveal the influence of load current on optical-fiber temperature, the currents of the power-unit and control unit conductors were gradually increased to 295 A and 63 A, respectively, under an ambient temperature of 20 °C. The second fiber-optic cable structure was selected as the analysis object, and the variation in internal optical-fiber temperature under straight-line and bending motions was investigated. The results are shown in Figs 11 and 12. The current step size and corresponding temperature data are shown in Table 7.

**Table 7:** Optical-fiber temperatures of fiber-optic shearer cable structure under different load currents and motion states.

Load level (%)	Power-unit current (A)	Control-unit current (A)	Optical-fiber temperature under straight-line motion (°C)	Optical-fiber temperature under bending motion (°C)
0	0	0	20.0	20.0
5	14.8	3.2	20.1	20.3
10	29.5	6.3	20.5	20.7
15	44.3	9.5	21.4	21.0
20	59.0	12.6	22.2	21.8
25	73.8	15.8	23.1	22.8
30	88.5	18.9	24.2	24.1
35	103.3	22.1	25.5	25.6
40	118.0	25.2	27.0	27.3
45	132.8	28.4	28.4	29.2
50	147.5	31.5	30.5	31.3
55	162.3	34.7	32.4	33.7
60	177.0	37.8	34.6	36.3
65	191.8	41.0	36.9	39.2
70	206.5	44.1	39.4	42.2
75	221.3	47.3	42.1	45.5
80	236.0	50.4	45.0	49.1
85	250.8	53.6	48.0	52.8
90	265.5	56.7	51.2	56.8
95	280.3	59.9	54.6	61.0
100	295.0	63.0	58.1	70.1

As shown in Table 7, the optical-fiber temperatures under both straight-line and bending motions generally increase nonlinearly with increasing load current. At load levels of 15%, 20%, 25%, and 30%, the optical-fiber temperatures under bending motion are slightly lower than those under straight-line motion, with differences ranging from 0.1 °C to 0.4 °C. At most other load levels, particularly under moderate and high loads, the optical-fiber temperature under bending motion is higher than that under straight-line motion. When the currents of the power-unit and control unit conductors reach 295 A and 63 A, respectively, the maximum optical-fiber temperatures under straight-line and bending motions are 58.1 °C and 70.1 °C, respectively. These results indicate that the influence of motion state is relatively limited at low load levels but becomes more pronounced as the load current increases.

### 3.4. Effect of ambient temperature on optical-fiber temperature

**Fig 13:**
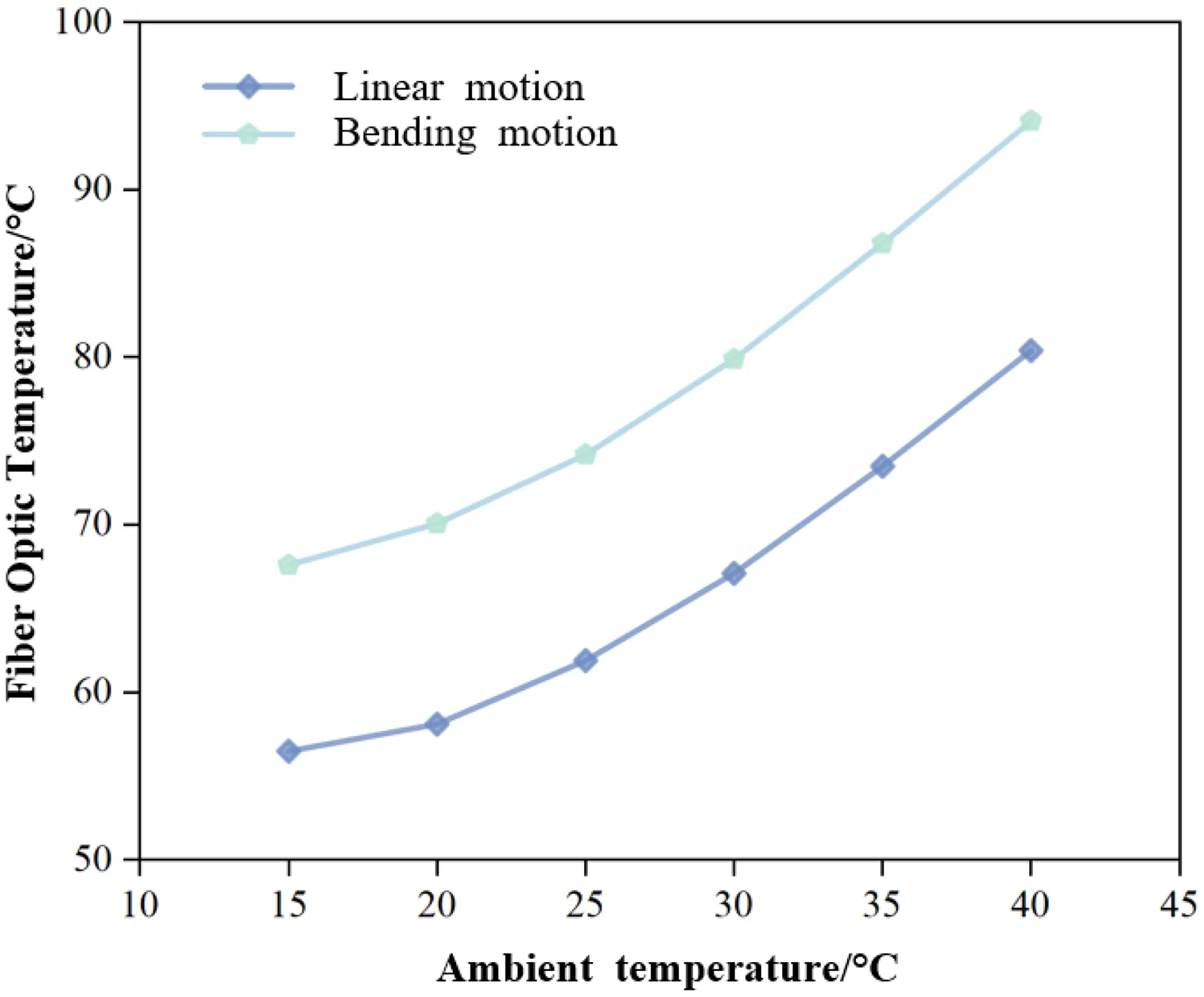
Variation curves of optical-fiber temperature with ambient temperature under straight-line and bending motions.

Based on the second fiber-optic shearer cable structure, the currents of the power-unit and control unit conductors were maintained at 295 A and 63 A, respectively. The ambient temperature was set within the range of 15–40 °C to analyze the variation of optical-fiber temperature under straight-line and bending motions. The simulation results under different ambient temperatures are listed in Table 8, and the optical-fiber temperature variation curves are shown in Fig 13. Under both straight-line and bending motions, the optical-fiber temperature increases significantly with increasing ambient temperature, and the rate of temperature rise gradually accelerates. When the ambient temperature reaches 40 °C, the maximum optical-fiber temperatures under straight-line and bending motions reach 80.4 °C and 94.1 °C, respectively. At the same ambient temperature, the optical-fiber temperature under bending motion is consistently higher than that under straight-line motion. Moreover, the temperature difference between the two motion states increases with increasing ambient temperature. This indicates that a high-temperature environment further amplifies the influence of the bending structure on optical-fiber temperature rise.

**Table 8:** Internal optical-fiber temperatures of the fiber-optic cable under different ambient temperatures.

Ambient temperature/°C	Optical-fiber temperature under straight-line motion/°C	Optical-fiber temperature under bending motion/°C
15	56.5	67.6
20	58.1	70.1
25	61.9	74.2
30	67.1	79.9
35	73.5	86.8
40	80.4	94.1

## 4. Relationship between the temperatures of internal cable structures and optical-fiber temperature

**Fig 14:**
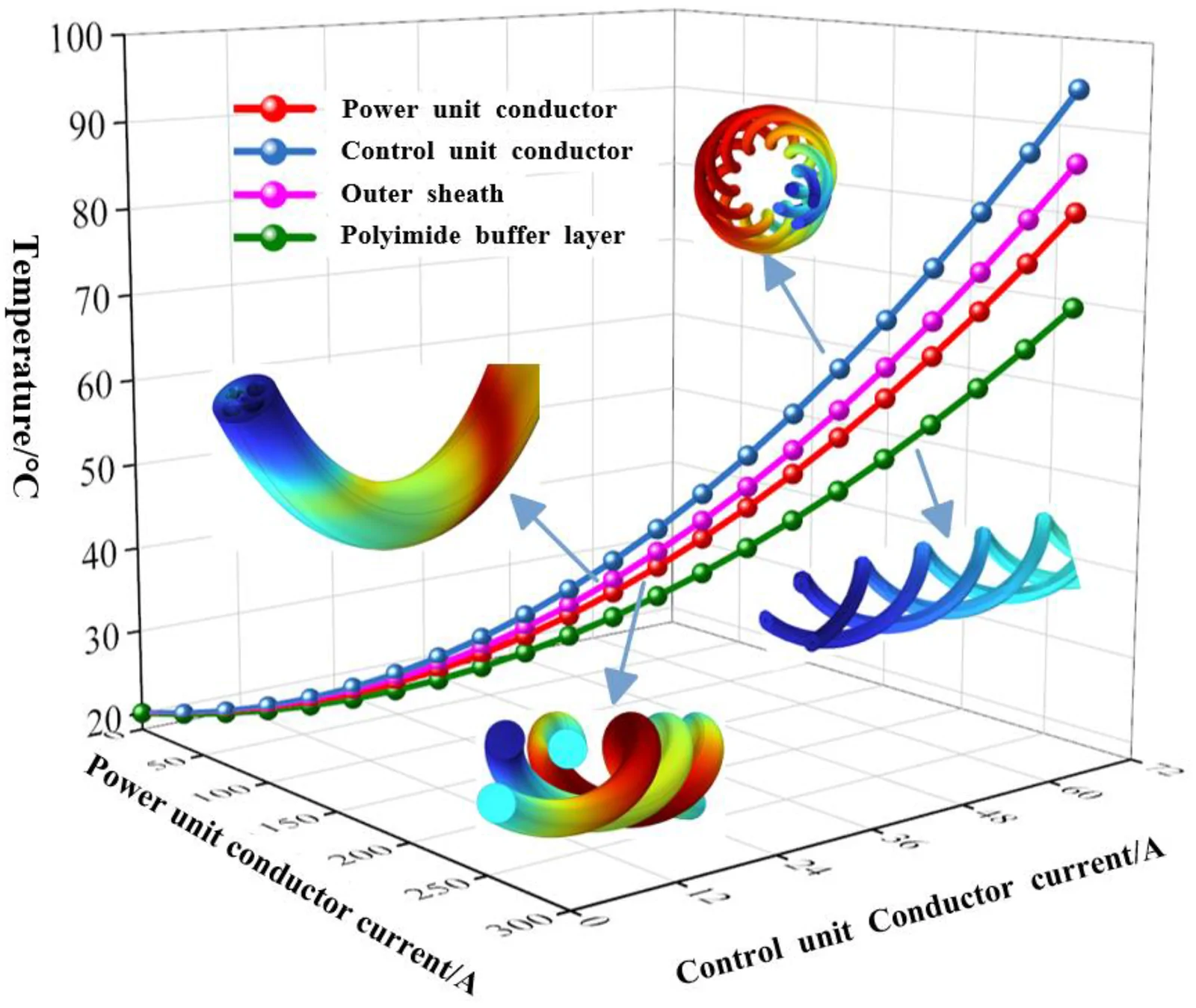
Temperature variation trends of different fiber-optic cable structures with load current at 20 °C.

The second fiber-optic shearer cable structure was selected for further analysis. The simulation ambient temperature was set to 20 °C, and the currents of the power-unit and control unit conductors were gradually increased to 295 A and 63 A, respectively. The temperature variations of the power unit conductor, control unit conductor, polyimide buffer layer, and outer sheath under bending motion were analyzed. The simulation results were extracted, and the temperature variation curves of different cable structures with load current at 20 °C are shown in Fig 14.

As shown in Fig 14, as the load current increases, the temperature of the internal conductor, polyimide buffer layer, and outer sheath of the optical cable gradually rises. When the currents of the power unit and control unit conductors increase to 295 A and 63 A, respectively, the temperatures of the power unit conductor, control unit conductor, outer sheath, and polyimide buffer layer reach 71.267 °C, 81.814 °C, 95.341 °C, and 87.194 °C, respectively. It can be seen that as the load current inside the conductor of the optical cable increases, the temperature of each cable component gradually increases, and the temperature rise and load current show a nonlinear growth trend.

Data for the power unit conductor, control unit conductor, polyimide buffer layer, outer sheath, and fiber optic temperature from the same set of simulation results were extracted separately, and the internal temperature of the cable at 20°C for each structure is shown in Table 9.

**Table 9:** Temperature table of internal structures of optical fiber cable at 20°C.

Fiber optic temperature (°C)	Power unit conductor temperature (°C)	Control unit conductor temperature (°C)	Outer sheath temperature (°C)	Polyimide buffer layer temperature (°C)
20	20	20	20	20
20.113	20.116	20.14	20.171	20.152
20.454	20.465	20.561	20.683	20.609
21.021	21.046	21.262	21.538	21.371
21.816	21.86	22.243	22.733	22.438
22.837	22.906	23.504	24.271	23.809
24.086	24.185	25.046	26.15	25.485
25.561	25.696	26.868	28.371	27.466
27.264	27.44	28.971	30.934	29.751
29.193	29.416	31.354	33.838	32.342
31.349	31.625	34.017	37.084	35.237
33.733	34.066	36.96	40.672	38.436
36.343	36.74	40.184	44.601	41.941
39.18	39.647	43.688	48.872	45.75
42.245	42.785	47.473	53.485	49.864
45.536	46.157	51.538	58.439	54.283
49.054	49.76	55.883	63.735	59.006
52.8	53.597	60.508	69.373	64.034
56.772	57.666	65.414	75.352	69.367
60.971	61.967	70.601	81.674	75.004
65.397	66.501	76.067	88.336	80.947
70.05	71.267	81.814	95.341	87.194

**Fig 15:**
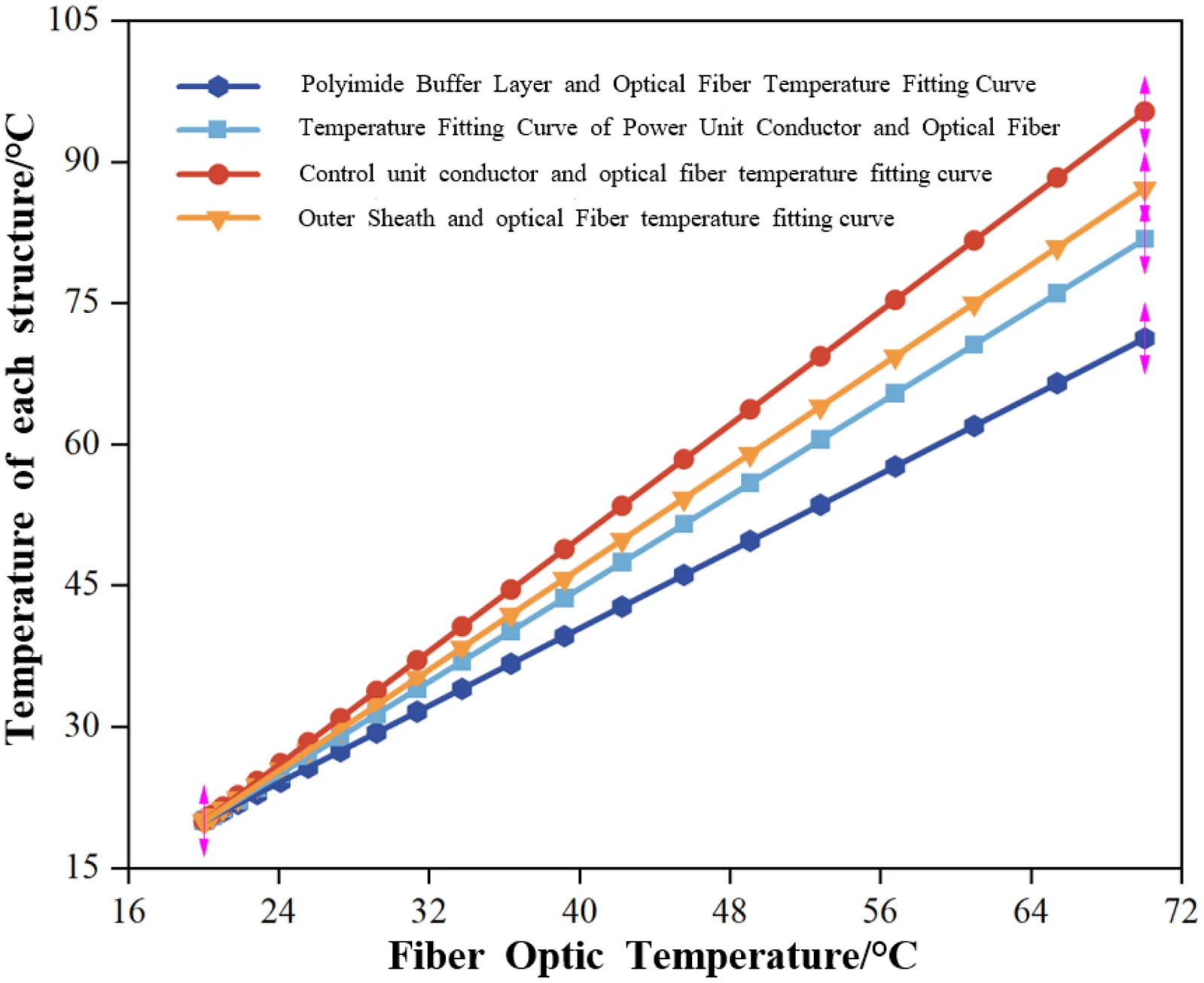
Fitted curves between optical-fiber temperature and the temperatures of different cable structures.

As shown in Table 9, the temperatures of the power-unit conductor, control-unit conductor, outer sheath, and polyimide buffer layer increase with the optical-fiber temperature. To quantify these relationships, the optical-fiber temperature was taken as the independent variable, while the temperatures of the four cable components were taken as dependent variables. Least-squares regression was then performed using the data listed in Table 9. To quantify the relationships between the optical-fiber temperature and the temperatures of the key cable components, the optical-fiber temperature was taken as the independent variable, while the temperatures of the power unit conductor, control unit conductor, outer sheath, and polyimide buffer layer were taken as dependent variables. Least-squares regression was then performed using the data listed in Table 9. The fitting curves of fiber temperature and temperature of each structure are shown in Fig 15.

(1) The relationship between fiber temperature and the conductor temperature of the control unit is

$$\begin{array}{c} T_{C}=1.235T_{f}-4.701 \end{array}$$ (10)

In the formula, *T*_*c*_ is the conductor temperature of the control unit, °C.

(2) The relationship between fiber temperature and outer sheath temperature is

$$\begin{array}{c} T_{CR}=1.505T_{f}-10.105 \end{array}$$ (11)

In the formula, *T*_*CR*_ is the outer sheath temperature, °C.

(3) The relationship between fiber temperature and the conductor temperature of the power unit is

$$\begin{array}{c} T_{P}=1.024T_{f}-0.486 \end{array}$$ (12)

In the formula, *T*_*p*_ is the conductor temperature of the power unit, °C; *T*_*f*_ is the fiber temperature, °C.

(4) The relationship between fiber temperature and polyimide buffer layer temperature is

$$\begin{array}{c} T_{PI}=1.342T_{f}-6.851 \end{array}$$ (13)

In the formula, *T*_*PI*_ is the temperature of the polyimide buffer layer, °C.

As indicated by the fitting results in Fig 15, the temperatures of the power unit conductor, control unit conductor, polyimide buffer layer, and outer sheath exhibit strong linear relationships with the optical-fiber temperature. These results provide a theoretical basis for real-time monitoring of the temperatures of different structures in fiber-optic cables using optical-fiber temperature measurements.

## 5. Discussion

The present study extends previous investigations on the thermal behavior of fiber-optic composite cables by focusing on fiber-optic shearer cables operating under the typical motion conditions of underground mining equipment. Previous studies have mainly investigated submarine power cables, optical-fiber composite low-voltage cables, and conventional power cables from the perspectives of electrothermal coupling, structural optimization, distributed optical-fiber sensing, and temperature prediction [2–11]. Compared with these studies, the present work considers not only the electrothermal response of the cable but also the influence of optical-fiber unit arrangement and cable motion state, which are particularly important for shearer cables subjected to repeated dragging and bending.

The simulation results show that the placement position of the optical-fiber unit has a much greater influence on optical-fiber temperature than the internal arrangement of the fibers. When the optical-fiber unit was located at the cable center, changing the fiber arrangement from a 1 + 3 parallel structure to a 1 + 3 stranded structure caused only a small change in the maximum optical-fiber temperature under both straight-line and bending motions. However, when the optical-fiber unit was cabled together with the control unit conductor, the maximum optical-fiber temperature increased significantly. This finding is consistent with the conclusions of Ashfaq et al. [3], Fu et al. [5], and Chen et al. [6], who reported that the position of the optical unit and the heat-resistant structural design strongly affect the thermal response of optical-fiber composite cables. In contrast to those studies, which mainly focused on low-voltage optical-fiber composite cables, the present study demonstrates that optical-fiber placement is also a dominant factor in the thermal design of shearer cables with complex helical structures.

The influence of load current observed in this study is also consistent with previous electrothermal analyses of power cables. The optical-fiber temperature increased nonlinearly as the power-unit and control-unit currents increased, which agrees with the general Joule-heating mechanism reported in earlier finite element studies [2,3,5]. Similar current-dependent temperature behavior was also considered by Zhao et al. [11] in the temperature prediction of shearer cables using a bidirectional long short-term memory model. However, the present study further reveals that, under most moderate- and high-load conditions, the optical-fiber temperature in the bending configuration is higher than that in the straight-line configuration. At several low-load levels, however, the differences are small and slight reversals occur. This result indicates that cable motion state should be considered as an additional thermal risk factor when evaluating the operating safety of shearer cables.

The effect of ambient temperature obtained in this study is in agreement with previous research on cable thermal behavior and optical-fiber temperature monitoring [7–11]. As the ambient temperature increased from 15°C to 40°C, the optical-fiber temperature increased continuously under both straight-line and bending motions. Moreover, the temperature difference between the two motion states became larger at higher ambient temperatures. This result suggests that high-temperature underground environments may amplify the thermal influence of bending deformation. Compared with the studies of Liu et al. [7] and Ti [9], which emphasized the use of distributed optical-fiber temperature sensing for fault diagnosis and thermal-state identification, the present work provides a structural and motion-state-based explanation for the measured optical-fiber temperature variation in shearer cables.

Another important result of this study is the fitted relationship between optical-fiber temperature and the temperatures of different internal cable structures. Previous studies, such as González-Cagigal et al. [4] and Li et al. [10], established relationships between optical-fiber temperature and conductor temperature in submarine cable systems using thermal network or numerical models. The present study obtains similar temperature-mapping relationships for fiber-optic shearer cables and further includes the power unit conductor, control unit conductor, polyimide buffer layer, and outer sheath. Therefore, the fitted linear relationships provide a useful basis for estimating the temperature of key internal structures from optical-fiber temperature measurements in shearer cables.

Overall, the present findings are consistent with previous studies in confirming that current load, ambient temperature, optical-fiber position, and thermal conduction paths are key factors affecting cable temperature. The main contribution of this study is that it connects these thermal factors with the special structural features and motion states of fiber-optic shearer cables. The comparison between straight-line and bending motions indicates that the influence of bending depends on the load level. At several low-load levels, the temperature differences between the two motion states are small, and the optical-fiber temperature under bending motion is slightly lower than that under straight-line motion. However, under most moderate- and high-load conditions, bending motion produces a higher optical-fiber temperature, indicating that the temperature difference between the two configurations becomes more pronounced as the load current increases. This result highlights the necessity of considering dynamic service conditions when designing fiber-optic shearer cables and interpreting optical-fiber temperature-monitoring data.

## 6. Conclusions

In this study, three fiber-optic shearer cable models were designed based on an MCPT-1.9/3.3(3 × 120 + 1 × 70 + 4 × 10) shearer cable. The effects of cable structure, load current, and ambient temperature on the internal optical-fiber temperature were investigated under straight-line and bending motions. Finally, functional relationships between the optical-fiber temperature and the temperatures of different cable structures were fitted. The main conclusions are as follows.

(1) Optical-fiber units were designed and embedded into a shearer cable to construct three fiber-optic shearer cable models. The influence of different structures on optical-fiber temperature was investigated. Numerical simulations of the three fiber-optic shearer cables under straight-line and bending motions were conducted using COMSOL. The results show that, at an ambient temperature of 20 °C and with the currents of the power-unit and control unit conductors set to 295 A and 63 A, respectively, the maximum optical-fiber temperatures of the three structures under straight-line motion are 57.8 °C, 58.1 °C, and 63.8 °C, respectively. Under bending motion, the corresponding temperatures are 69.5 °C, 70.1 °C, and 77.4 °C. The temperature difference between the first and second structures is small, whereas the third structure exhibits a significantly higher temperature, indicating that the optical-fiber unit position is the primary factor affecting temperature rise. Under the rated-load condition, the optical-fiber temperatures of the three curved cable configurations were 11.7 °C, 12.0 °C, and 13.6 °C higher than those of the corresponding straight-line configurations, respectively. These differences reflect the influence of cable geometry on the electrothermal field.
(2) The second fiber-optic shearer cable structure was selected for further investigation. Under both straight-line and bending motions, the optical-fiber temperature generally increases nonlinearly with increasing load current. At several low-load levels, the optical-fiber temperature under bending motion is slightly lower than that under straight-line motion, with differences of only 0.1–0.4 °C. Under most moderate- and high-load conditions, however, the bending temperature is higher. When the currents of the power-unit and control unit conductors reach 295 A and 63 A, respectively, the optical-fiber temperatures under straight-line and bending motions reach 58.1 °C and 70.1 °C, respectively, with the bending temperature being 12.0 °C higher. This indicates that the thermal influence of bending becomes more evident as the load current increases. When the load current remains constant, the optical-fiber temperature increases with increasing ambient temperature. At an ambient temperature of 40 °C, the optical-fiber temperatures under straight-line and bending motions reach 80.4 °C and 94.1 °C, respectively, with the bending state being 13.7 °C higher.
(3) The least-squares method was used to fit the temperature data of the power unit conductor, control unit conductor, polyimide buffer layer, outer sheath, and optical fiber. The results show that the temperatures of the internal structures of the fiber-optic cable have strong linear relationships with the optical-fiber temperature. This provides a theoretical basis for real-time monitoring of the temperatures of different fiber-optic cable structures using optical fibers.
